# Utility of Vascular Endothelial Growth Factor Inhibitors in the Treatment of Ovarian Cancer: From Concept to Application

**DOI:** 10.1155/2012/540791

**Published:** 2011-09-26

**Authors:** Afshin Amini, Samar Masoumi Moghaddam, David L. Morris, Mohammad H. Pourgholami

**Affiliations:** ^1^Cancer Research Laboratories, Department of Surgery, St George Hospital (SESIAHS), The University of New South Wales, Sydney, NSW 2217, Australia; ^2^Department of Surgery, St George Hospital (SESIAHS), The University of New South Wales, Sydney, NSW 2217, Australia

## Abstract

Despite recent advances in the management of ovarian cancer, it remains the most lethal gynecologic malignancy. Vascular endothelial growth factor (VEGF) has been shown to play a pivotal role in the progression of ovarian cancer leading to the eventual development of malignant ascites. On this basis, agents rendering VEGF ineffective by neutralizing VEGF (bevacizumab), blocking its receptors (aflibercept), or interfering with the postreceptor signaling pathways (sunitinib) provide us with the rational treatment options. These agents are generally used in combination with the standard chemotherapeutic drugs. Here, we discuss the basis of and the logic behind the use of these agents in the treatment of epithelial ovarian cancer, as well as their evaluation in different preclinical and clinical studies.

## 1. Introduction

Ovarian cancer is the sixth leading cancer diagnosed among women in the world and the second most common gynecologic cancer, comprising nearly 4% of all female cancers [1, 2]. Due to lacking early warning signs and effective screening tools, approximately 75% of patients present with late stage disease [3].

Conventional cytotoxic chemotherapy induces a large proportion of responses in the first-line treatment of advanced ovarian cancer with response rates of 60–80%, but the subsequent relapse and death will develop in the majority of patients [4]. Most cases of advanced ovarian cancer will show resistance to chemotherapy in spite of intensive primary interventions. Targeting tumor vasculature has been theoretically a focus of great interest in this regard due to not only its vital role of conducting tumor blood supply, but also its epithelial cells being more genetically stable than tumor cells [5]. 

Reviewing the role of vascular endothelial growth factor in ovarian cancer and the feasibility and possible role of VEGF-targeted strategies in ovarian cancer treatment as well as their promises and challenges is the aim of this article.

## 2. The Role of Angiogenesis in Ovarian Physiology

Female reproductive cycle is intricately connected to the coordinated action of angiogenic factors and steroid hormones. Dominant follicles starting maturation in ovulatory cycle are those with higher vascularity that eventually synthesize the steroid hormones required for endometrial development by turning into corpus luteum. This increased vascularity continues during luteal phase to supply nutrients and steroid precursors and helps to make active steroid hormones accessible to the endometrium. Cyclical changes in the level of vascular growth factors in different stages of menstrual cycle indicate the importance of angiogenesis in ovarian physiology. Increased intrafollicular levels of VEGF have been shown during the initial part of the ovulatory cycle, with peak concentrations just before the start of the luteal phase [6]. 

## 3. Tumor Angiogenesis and Its Role in Ovarian Cancer

Like their normal counterparts, tumor cells are in crucial need of a vascular system to satisfy their own requirements of having access to oxygen and nutrients supply and waste removal. Tumor angiogenesis is the mechanism required for fulfilling these requirements, without which tumors fail to grow beyond 1-2 mm and may remain dormant [7]. 

Tumor-induced blood vessels possess ultrastructural abnormalities, including lack of functional pericytes, dilation and convolution, exceptional permeability, and vascular walls being infiltrated by tumor cells [8].

In ovarian cancer, an imbalance between tumor levels of pro- and antiangiogenic factors in favor of angiogenesis activation occurs. It is indicated as increased proangiogenic factors, including VEGF, fibroblast growth factor (FGF), platelet-derived growth factors (PDGFs), tumor necrosis factor-alpha (TNF-*α*), angiopoietins and interleukins (IL-6, IL-8), and decreased antiangiogenic factors, including angiostatins and endostatins [9]. It has been indicated that the modulation of angiogenic pathways in ovarian surface epithelium may alter its tumorigenicity [10]. Although microvessel density (MVD) in ovarian cancer has correlated with extent of disease and, inversely, with overall survival (OS) or progression free survival (PFS) [11], different studies have reported contradictory data regarding a convincing correlation between MVD and ovarian cancer prognosis [12–14].

## 4. The Role of Vascular Endothelial Growth Factor in Ovarian Cancer

VEGF promotes proliferation, migration, stabilization, and survival of endothelial cells and mobilization of endothelial progenitor cells from bone marrow and yields a direct effect on tumor cell proliferation and invasiveness as well [9, 15]. 

VEGF, formerly known as vascular permeability factor (VPF), has also a key role in enhancing vascular permeability [9]. In advanced ovarian cancer, VEGF-induced hyperpermeability of peritoneal blood vessels and subsequent intraperitoneal hyperosmolarity caused by leaked plasma proteins will lead to malignant ascites, a prevalent, debilitating manifestation of the late-stage disease indicating disease progression and treatment failure [16]. Moreover, some leaked proteins such as plasminogen activator, matrix metalloproteinases, interstitial collagenases, and gelatinase-A provide space for new cell growth through degrading extracellular matrix while fibrinogen facilitates microvascular growth [15]. 

VEGF induces tumor angiogenesis in a way very similar to how it promotes physiological angiogenesis [17]. As a mitogen for vascular endothelial cells, VEGF promotes new blood vessels formation, and as a survival factor, it stabilizes new, poorly formed tumor vasculature and inhibits endothelial cell apoptosis, resulting in sustained tumor growth [15]. It has been demonstrated that VEGF inhibition normalizes tumor vessels and enhances oxygen and chemotherapeutics delivery to the tumoral tissue [6].

Investigations show that VEGF also contributes to tumor metastasis by inducing the formation of structurally abnormal blood vessels that can be easily penetrated by neoplastic cells [17]. Moreover, increased expression of matrix metalloproteinase-2 by VEGF can enhance the invasiveness of tumor cells [9].

In addition to its paracrine effect on tumor vascular endothelial cells, VEGF has been found to have an autocrine effect by interesting discovery of VEGF receptors on tumor cells [18, 19]. However, the functional significance of this finding and the underlying cellular processes of VEGF/VEGFR autocrine loop are being studied. A functional role for VEGFR-2 and a distinct VEGFR-2-mediated pathway promoting tumor growth in ovarian cancer have been demonstrated [20].

VEGF expression in ovarian cancer has been evaluated in several studies. Some degree of VEGF expression in all examined ovarian cancer specimens as well as significantly higher levels of VEGF expression in tumor specimens compared to benign ovarian tissue have been reported [21]. A correlation between increased titers of VEGF in cytosolic fractions from tumor specimens and increased stage and decreased survival was found as well. In early stage ovarian cancers, increased VEGF expression has been shown to correlate with worse disease-free survival (DFS) and poor OS [22]. In addition, higher serum levels of VEGF associated with ovarian cancer were considered as an independent risk factor and a prognostic parameter for ascites, more metastasis, advanced-stage disease, and decreased survival [23, 24]. VEGF upregulation enhanced the invasiveness of ovarian cancer cells in vitro [25] and VEGF blockade in animal models of ovarian cancer inhibited ascites formation and slowed the tumor growth [26]. Several retrospective clinical studies in ovarian cancer have also demonstrated that intratumoral VEGF and VEGFR-2 expression and VEGF gene polymorphisms are independent poor prognostic factors [27–29]. Overexpression of neuropilin, a coreceptor enhancing VEGF signaling, has also been found in ovarian cancer [30, 31].

## 5. Anti-VEGF Agents in Ovarian Cancer: Previous and Current Studies

Known to play a key role in normal ovarian physiology and in ovarian cancer, VEGF signaling axis has been an attractive target in antiangiogenic approaches. Agents that target this pathway are currently in clinical development for ovarian cancer (Table 1).

### 5.1. VEGF Ligand Binders

#### 5.1.1. Bevacizumab

Bevacizumab is a recombinant humanized VEGF monoclonal antibody derived from its murine equivalent A4.6.1. It directs against all active isoforms of VEGF and prevents them from binding to VEGFR [32]. Known as the first anti-VEGF agent approved by the Food and Drug Administration (FDA) in 2004 for clinical use in colorectal cancer, bevacizumab has also been the first anti-VEGF agent to be evaluated in the treatment of ovarian cancer [4, 33].

**Preclinical Data**

Several experiments have shown that neutralizing VEGF by bevacizumab has marked antitumor effects. In 1998, Mesiano et al. examined the role of bevacizumab in immunodeficient mice with ovarian cancer and demonstrated that bevacizumab significantly inhibited subcutaneous tumor growth, partially inhibited its intraperitoneal growth, and completely prevented ascites production [34]. Hu et al. reported additive or synergistic effects of this antibody in combination with paclitaxel in ovarian tumor xenograft studies including enhanced sensitivity to paclitaxel and marked reduction of tumor growth and ascites formation [35]. Those effects have been confirmed by Mabuchi et al. using bevacizumab in combination with cisplatin. Moreover, they showed that maintenance treatment with bevacizumab could inhibit recurrence and significantly prolong survival in vivo [36].

**Clinical Data**

 

**(i) As a Single Agent**

The initial clinical evidence regarding the activity of bevacizumab for ovarian cancer, mainly in recurrent, heavily pretreated patients, was first reported by Monk et al. in 2005 [42]. Since then, bevacizumab has been examined in two prospective phase II trials as a single agent for patients with recurrent ovarian cancer, predominantly platinum-resistant disease as shown in Table 2 [37, 38].

**(ii) Combined with Chemotherapy**

 

**Retrospective Studies**

In 2006, Wright et al. reported a partial response (PR) of 35% and a median PFS of 5.6 months in a retrospective analysis of 23 patients with recurrent platinum-refractory epithelial ovarian cancer treated with a combination of bevacizumab with some cytotoxic agents including cyclophosphamide, 5-fluorouracil, docetaxel, and gemcitabine and liposomal doxorubicin [43]. In another study on 35 patients with recurrent ovarian cancer who received a combination of gemcitabine, platinum, and bevacizumab, Richardson et al. reported an overall response rate (ORR) of 78%, a complete response (CR) of 48%, and a median PFS of 12 months [44]. In a study by Chura et al. on 15 heavily pretreated patients with recurrent ovarian cancer, a combination of bevacizumab and metronomic oral cyclophosphamide resulted in a CR of 13.3% and a PR of 40% [45]. O'Malley and colleagues have recently reported the results of a study on two groups of heavily pretreated patients with recurrent ovarian cancer: 29 patients treated with weekly paclitaxel as compared to 41 patients treated with weekly paclitaxel and biweekly bevacizumab. They have indicated that addition of bevacizumab to weekly paclitaxel has resulted in a significant increase in PFS with a trend towards improved OS [46].

**Clinical Trials**

To date, three clinical trials on bevacizumab combined with chemotherapy have been published. A summary of the outcome from these studies is listed in Table 3 [39–41].

Bevacizumab is currently being evaluated in combination with various chemotherapy regimens briefly described in Table 4.

The results from GOG218 (NCT00262847) have been reported at the 2010 American Society of Clinical Oncology (ASCO) annual meeting. Based on their report, there was a 3.8-month improvement in PFS (14.1 months for the maintenance regimen of BEV as opposed to 10.3 months for standard chemotherapy) [47]. Initial results of ICON7 (NCT00483782) trial presented in October 2010 showed that the addition of bevacizumab to standard chemotherapy resulted in 15% improvement in PFS at 12 months, 1.7-month improvement in median PFS, and 1.5-month overall improvement in PFS. Treatment effect is numerically greater in advanced-stage patients with no new side effects [48].

**Side Effects**

Being generally well tolerated, the most common bevacizumab-attributable side effect, hypertension, can be medically controlled. However, gastrointestinal perforation and thromboembolic disease were documented as two major complications [33], and the overall rate of bowel perforation in ovarian cancer seems to be higher than other solid tumors. Other common adverse events include proteinuria, bleeding, and wound-healing complications [9]. Preliminary analysis of a prospective study evaluating the safety and efficacy of bevacizumab in cancer patients has demonstrated severe pulmonary and nonpulmonary hemorrhage as an associated risk with a rate of 0.5% and 1.2%, respectively [49]. Also, a meta-analysis of randomized controlled trials has recently reported that bevacizumab may significantly increase the risk of serious hemorrhage in cancer patients [50]. Reversible posterior leukoencephalopathy syndrome (RPLS), tracheoesophageal fistulae [6], spontaneous nasal septal perforation, and erosive osteoarthritis are significant, but rare, complications [9]. In a recent meta-analysis of published randomized controlled trials, bevacizumab in combination with chemotherapy or biological therapy, compared with chemotherapy alone, was associated with increased treatment-related mortality [51].

#### 5.1.2. Aflibercept (VEGF Trap)

Aflibercept is a soluble decoy receptor based on VEGF receptor-1 and VEGF receptor-2 fused to the Fc portion of human immunoglobulin G1 that binds and inactivates some members of VEGF family, including VEGF-A, VEGF-B, and placental growth factor (PlGF) [4].

**Preclinical Data**

In 2003, Byrne et al. reported that single-agent aflibercept significantly reduced both ascites and tumor burden in experimental ovarian cancer models [26]. This preclinical efficacy was further validated by Hu et al. who showed a 98% reduction in tumor burden, inhibition of ascites, and prolonged survival in a mouse model of human ovarian cancer treated by aflibercept plus paclitaxel [52].

**Clinical Data**

Aflibercept has been investigated in some clinical studies described in Table 5.

**Side Effects**

Based on the initial results of their study, Tew et al. reported that the most common grade 3-4 side effects of aflibercept therapy were hypertension (9%) and proteinuria (4%). Other adverse events included headache, fatigue, dysphonia, nausea, asthenia, diarrhea, renal dysfunction, and a remarkably lower incidence of bowel perforation (1%) [53].

### 5.2. VEGF Receptor Tyrosine Kinase Inhibitors

#### 5.2.1. Ramucirumab (IMC-1121B)

Ramucirumab is a fully humanized monoclonal antibody that specifically and potently inhibits VEGFR-2 [9]. On the basis of in vitro studies, ramucirumab has been investigated in mouse xenograft models of human ovarian cancer resulting in reduced tumor growth, increased apoptosis, and decreased tumor microvessel proliferation and density [55]. Being observed in several phase I clinical trials in solid tumors [56], ramucirumab is currently being assessed in a phase II trial as a monotherapy in patients with persistent or recurrent epithelial ovarian cancer [57].

Ramucirumab has shown safety in phase I trials. Two dose-limiting toxicities including hypertension and deep vein thrombosis were noted by Spratlin et al. in a phase I trial [58].

#### 5.2.2. Cediranib (AZD2171)

This is a novel oral tyrosine kinase inhibitor that selectively blocks VEGFR1-3, PDGFR-*β*, and c-Kit [4]. Cediranib has been shown to inhibit the growth of human tumor xenografts, including ovarian cancer, in a dose-dependent manner [59]. In 2009, the results of a phase II study of single-agent cediranib for recurrent ovarian, peritoneal, or tubal cancer were reported by Matulonis et al. Overall clinical benefit for the intent-to-treat (ITT) population was 30%; 17% patients achieved a partial response representing the overall response rate. Thirteen percent of patients had stable disease. No patients had a complete response [60]. In another phase II trial by Hirte et al., response rate was 41% and 29% for platinum-sensitive and platinum-resistant disease, respectively [61]. Other phase II and III trials [62–64] are ongoing to investigate its efficacy as single agent or in combination therapy, among which is ICON6, a three-arm randomized placebo-controlled phase III trial, investigating cediranib in combination with platinum-based chemotherapy and as a single-agent maintenance therapy in patients with platinum sensitive relapsed ovarian cancer [64].

In both above-mentioned phase II trials, hypertension and fatigue were the most common grade 3 toxicities. Other reported adverse events were central nervous system hemorrhage, elevated lipase, hypertriglyceridemia, diarrhea, anorexia, vomiting, hyponatremia, oral cavity pain, nausea, constipation, abdominal pain, headache, and hypothyroidism [61, 65].

#### 5.2.3. Semaxanib (SU5416)

Potent and selective synthetic inhibitor of VEGFR-2, semaxanib inhibits tyrosine kinase catalysis, tumor vascularization, and growth of different tumor types [66]. In a study by Holtz et al., semaxanib yielded reduced tumor growth and microvessel density in mouse models of ovarian cancer with high VEGF expression. Based on its combination with metronomic paclitaxel, they also provided the first evidence that the interactions between low-dose chemotherapy and antiangiogenic therapy could be affected by tumor VEGF expression as they observed additive effects only in tumors with low VEGF expression [67]. A phase I study of semaxanib in combination with carboplatin in patients with platinum-refractory ovarian cancer has been done [68].

### 5.3. Multiple-Receptor Tyrosine Kinase Inhibitors

#### 5.3.1. Sunitinib (SU11248)

Sunitinib is an orally bioavailable multityrosine kinase inhibitor that blocks VEGFR1-3, Flt-3, PDGFR-*α*, PDGFR-*β*, c-Kit, CSF-1R, and RET with proven antitumor activity in renal cell carcinoma and imatinib-resistant or -intolerant gastrointestinal stromal tumor (GIST) [4]. Its effectiveness as a single agent in recurrent or advanced ovarian cancer was proved by Bauerschlag et al. in an ovarian cancer xenograft mouse model, in which the drug significantly suppressed tumor growth and peritoneal metastases, and also remarkably reduced microvessel density count [69].

Having similar gene profile to renal cell carcinoma (RCC), recurrent or refractory ovarian clear cell adenocarcinoma—a biological subtype of epithelial ovarian cancer—may benefit from this agent. In one case report of a 60-year-old woman with ovarian clear cell adenocarcinoma (OCCA), sunitinib resulted in stable disease as fifth-line therapy, decreased CA125 and cystic degeneration of liver metastasis [70]. Based on the promise shown in different phase I, II, and III studies of sunitinib in a number of cancers, four phase II trials of sunitinib as single-agent therapy in ovarian cancer are being pursued [71–74]. One of those is NCT00388037, first results of which have demonstrated single-agent sunitinib activity and tolerability in advanced ovarian cancer with partial response in 2 and stable disease in 10 among 17 patients [74].

Both on-target and off-target adverse effects including fatigue, diarrhea, dyspepsia, hypertension, hand-foot syndrome, nausea, anorexia, stomatitis, neutropenia, thrombocytopenia, lipase elevations, and hypothyroidism are mentioned as typical side effects of sunitinib in other diseases [9, 75].

#### 5.3.2. Sorafenib (Bay43-9006)

Sorafenib is an oral multitargeted tyrosine kinase inhibitor that predominantly inhibits Raf-1—which is vital for cell proliferation—and can block VEGFR1-3, PDGFR-*β*, Flt-3, and c-Kit [9]. In a study by Matsumura et al., sorafenib was shown to have antitumor effect against ovarian clear cell carcinoma (OCCC) as it inhibited tumor growth in nude mice and significantly reduced tumor size [76]. In a phase I study of sorafenib in patients with ovarian cancer, 50% of them showed evidence of stable disease [77]. In another phase I trial, sorafenib in combination with bevacizumab demonstrated durable partial disease responses in 6 of 13 ovarian cancer patients recruited [6]. In a phase II trial in patients suffering from persistent or recurrent ovarian cancer, partial response was seen in 3% of patient evaluated while 20% had stable disease more than 6 months [78]. Another study of intermittent sorafenib dosing with bevacizumab has promising clinical activity and less sorafenib dose reduction and side effects in advanced ovarian cancer [79]. Six clinical trials of sorafenib combined with other agents are now underway [80–85].

Although diarrhea and hand-foot syndrome are more prevalent than other side effects, alopecia, anorexia, and weight loss also have been reported more common with sorafenib. However, few grade 3 and 4 adverse effects have occurred [75].

#### 5.3.3. Vatalanib (PTK787)

Being a multitargeted tyrosine kinase inhibitor, vatalanib blocks VEGFR1-3, PDGFR-*β*, c-Kit, c-Fms with highest activity against VEGFR-2. In an ovarian cancer mouse model, single-agent vatalanib reduced ascites and tumor growth and yielded increased survival [15]. In a phase I study of vatalanib combined with carboplatin and paclitaxel in advanced ovarian cancer, Schroder et al. showed that vatalanib was feasible and well tolerated [86]. The side effects of vatalanib appear to be similar to those of other VEGF inhibitors. Schroder et al. reported grade 1 and 2 hypertension as the most frequent adverse events in their study. 

#### 5.3.4. Vandetanib (ZD6474)

Vandetanib is a dual specific inhibitor of VEGFR and EGFR. Monotherapy with vandetanib showed a significant antitumor effect in an ovarian cancer nude mice model [87]. No significant clinical benefit was made by vandetanib monotherapy in patients with recurrent ovarian cancer in a phase II clinical trial [88]. Its efficacy in combination with docetaxel in persistent or recurrent ovarian cancer will be assessed in a phase II clinical trial (NCT00872989) [89]. Common drug-related adverse events include rash, diarrhea, hypertension, fatigue, and asymptomatic QTc prolongation [90].

#### 5.3.5. Intedanib (BIBF1120)

Intedanib is a combined inhibitor of VEGFR, PDGFR, and FGFR. Having potential to block proangiogenic signaling pathways in vascular endothelial cells, smooth muscle cells, and pericytes, indetanib can inhibit cell proliferation and apoptosis. In a study by Hilberg et al., indetanib demonstrated high activity at well-tolerated dose as decreased vessel density and vessel integrity, and profound growth inhibition in all tested tumor models [91]. Ledermann et al. tested indetanib as maintenance therapy in a randomized phase II placebo-controlled trial in ovarian cancer patients who had previously responded to chemotherapy and resulted in a 36-week PFS rate of 15.6% and 2.9%, respectively, for indetanib and placebo. Thus, the conclusion that maintenance indetanib could delay disease progression in previously treated ovarian cancer patient was drawn [92]. To investigate its efficacy and safety, indetanib combined with carboplatin and paclitaxel is currently being examined in a randomized, double-blind phase III trial in patients with advanced ovarian cancer [93]. Based on Ledermann and colleagues' study, the rate of gastrointestinal toxicities was slightly higher in the indetanib arm. They also found higher elevation of liver enzymes (43%) compared with placebo (6.3%) [92]. 

#### 5.3.6. AEE788

AEE788 is a combined VEGFR and EGFR specific inhibitor. Although single-agent AEE788 was effective in reducing tumor weight in a nude mice model of human ovarian cancer, the combination of AEE788 and paclitaxel was superior to the use of either agent alone, inhibiting the progression of intraperitoneal tumor [94]. Encouraging activity was also indicated by metronomic docetaxel chemotherapy combined with AEE788 in orthotopic mouse model resistant to the conventional therapy [6, 95].

#### 5.3.7. Pazopanib

Pazopanib is a potent and selective multitargeted receptor tyrosine kinase inhibitor of VEGFR, PDGFR, and c-Kit. Pazopanib inhibits VEGF-induced endothelial cell proliferation in vitro and angiogenesis in vivo and shows antitumor activity in mouse models [96]. Pazopanib alone and combined with metronomic oral topotecan was tested by Merritt et al. in vitro and in an orthotopic model of ovarian cancer. Pazopanib therapy resulted in reduced murine endothelial cell migration in a dose-dependent manner and decreased tumor cell proliferation. Combination therapy increased tumor cell apoptosis and reduced tumor microvessel density and pericyte coverage [97]. A phase II study reported by Friedlander et al. indicated that pazopanib is active in women with advanced epithelial ovarian cancer with overall response rate of 18% and 21%, respectively, in subjects with and without measurable disease at baseline. Median PFS was 84 days [98]. Several trials of pazopanib are evaluating this agent as monotherapy or combination with other agents [99–103].

#### 5.3.8. Motesanib (AMG706)

Motesanib was identified as a potent, well-tolerated inhibitor of VEGFR 1–3 and PDGFR in preclinical models. It also inhibits Kit receptors, thereby directly interfering with signal transduction of the tumor cell [104]. A phase II clinical trial of this agent in persistent or recurrent ovarian cancer is ongoing [105].

## 6. Other Agents

Some agents from different pharmacological classes have been studied in clinical development for ovarian cancer with possible or indirect effect on VEGF or its pathway. 

### 6.1. Thalidomide

Thalidomide was first introduced as a sedative drug in the late 1950s. In 1961, it was withdrawn from the market due to its teratogenicity [106, 107], but it has been of renewed interest as a potent angiogenesis inhibitor which was approved by FDA [75]. Antiangiogenic and antitumor effects of thalidomide have been demonstrated in preclinical studies [108, 109], although its mechanisms of action are not clearly unveiled. Thalidomide may suppress VEGF, bFGF, and TNF-*α*. A phase II trial of thalidomide in some solid malignancies, including ovarian cancer, resulted in some responses whereas a prospective randomized trial of thalidomide with topotecan compared to topotecan alone in women with recurrent epithelial ovarian carcinoma reported a remarkable response with combination therapy [5]. A randomized phase III trial (GOG 198) tested tamoxifen versus thalidomide in women having the recurrence of ovarian cancer. Thalidomide was not more effective than tamoxifen in delaying recurrence or death, but was more toxic [110].

### 6.2. Atrasentan

Atrasentan is a selective antagonist of endothelin type A receptor (ETAR). It reduces microvessel density, expression of VEGF, and matrix metalloproteinase-2, thus increases percentage of apoptotic tumor cells in ovarian cancer xenografts. Combined with paclitaxel, atrasentan has produced additive antitumor, apoptotic, and antiangiogenic effects. Fatigue, edema, and rhinitis are the most common side effects of atrasentan [9].

### 6.3. Everolimus

Everolimus is an inhibitor of mTOR, a central regulating pathway of cell growth, proliferation, and apoptosis. Inhibition of mTOR can decrease cancer cell proliferation and survival and reduce tumor-secreted VEGF through inhibition of hypoxia-inducible factor-1**α** (HIF-1**α**). An in vivo study using xenograft models of ovarian cancer revealed that everolimus inhibited tumor growth, angiogenesis, and production of ascites, suggesting the potential of mTOR inhibitors in the treatment of women with ovarian cancer [9, 98].

### 6.4. Microtubule Disrupting Agents

Drugs that target tubulin are one of the most effective classes of anticancer agents and are thus a mainstay in the treatment of ovarian cancer [111]. Although the underlying mechanisms for the inhibition of angiogenesis by microtubule disrupting agents (MDAs) are not yet well defined, interference with the HIF-1*α*/VEGF axis seems to account for at least part, if not all, of the effects observed [112]. While the taxanes are defined as microtubule polymerizing agents, benzimidazole carbamates such as albendazole are known to conversely interfere with the polymerization process. We have recently described how albendazole inhibits VEGF and angiogenesis under in vitro, in vivo, and clinical conditions [113–115]. Follow-up research revealed that the drug interferes with HIF-1*α*, leading to the suppression of tumoral VEGF mRNA, and VEGF protein [116]. Thus, irrespective of whether a polymerizing or a depolymerising agent, the MDAs interfere with angiogenesis and suppress VEGF production. Treatment of mice bearing advanced intraperitoneal human OVCAR-3 tumors with albendazole, led to suppression of both plasma and ascites VEGF levels, as a consequence of which highly VEGF-dependant malignant ascites formation was completely aborted, leading to extended animal survival [115, 117]. 

### 6.5. Celecoxib and Ciglitazone 

PGE2 enhances angiogenesis through the induction of VEGF. Celecoxib is a highly selective cyclooxygenase-2 (COX-2) inhibitor and ciglitazone is a peroxysome proliferator-activated receptor **γ** (PPAR**γ**) ligand, both inhibiting prostaglandin E2 (PGE2) production. In a preclinical ovarian cancer model, celecoxib and ciglitazone reduced tumor growth by downregulating PGE2 synthesis and thus inhibiting VEGF production [9]. In a phase II study in heavily pretreated recurrent ovarian cancer patients, celecoxib in combination with carboplatin was well tolerated and yielded promising activity as salvage treatment [118]. Celecoxib is also being evaluated in an ongoing randomized phase II study testing cyclophosphamide with or without celecoxib [119].

## 7. Anti-VEGF Therapy Challenges

Besides toxicities and complications, other challenges regarding anti-VEGF therapy are as follows.

### 7.1. Resistance

Although endothelial cells are genetically stable, emerging evidence indicates that tumor resistance to anti-VEGF agents is common. The hypothetical rationale for the resistance includes epigenic mechanisms of resistance, unresponsiveness of tumor vasculature to anti-VEGF agents [5], upregulation of alternative proangiogenic pathways, enhanced protection by pericytes, increased invasiveness of tumor cells into local tissue normal vasculature, metastatic seeding, and tumor cell growth in lymph nodes and distant organs, as well as failure of anti-VEGF agents in fully blocking all VEGF signaling pathways [120, 121]. Locating additional targets on the tumor endothelium such as nonreceptor kinases, and targeting proangiogenic pathways and agents such as HIF1-*α* are currently being focused to evade the resistance [5]. 

### 7.2. Acceleration of Tumor Invasiveness and Metastasis

A perplexing inverse effect resulted from anti-VEGF therapy has been found in some preclinical studies. Ebos et al. reported:

- accelerated experimental metastasis, increased multiorgan metastases, and decreased survival after short-term sunitinib treatment before and after intravenous tumor cells inoculation;
- increased spontaneous metastasis and decreased survival following short-term sunitinib therapy after removal of primary human xenograft tumors.

Acceleration of metastasis observed in mice receiving sunitinib prior to intravenous implantation of tumor cells suggests the possible “metastatic conditioning” effect by the mentioned anti-VEGF agent. It means microenvironmental changes in mouse organs so that they are conditioned to be more permissive to tumor extravasation. This study shows similar findings with additional VEGF receptor tyrosine kinase inhibitors, implicating a class-specific effect for this group of anti-VEGF agents [122]. Also, Paez-Ribes et al. indicated the following:

- increased invasiveness and metastasis in tumor-bearing mice treated by the VEGFR2 blocking monoclonal antibody DC101;
- persistent invasive phenotype after cessation of the treatment.

Similar results were reported with sunitinib and SU10944 administration. Paez-Ribes et al. demonstrate that these effects appear to be an adaptive/evasive response by tumor cells themselves involving an augmented invasive phenotype and, in some cases, increased dissemination and the emergence of distant metastasis. They implicate hypoxia in the adaptive response [123].

These studies indicate divergent effects of anti-VEGF agents on primary tumor growth and metastasis and raise the possibility that both induction and suppression of tumor angiogenesis can exert proinvasive/prometastatic effects [122–124].

### 7.3. Pharmacoeconomics

As health care costs continue to increase, chemotherapy agents, and in particular targeted therapies, have been scrutinized regarding the populations in which they should be used to minimize the societal impact of their utility. Using an intentionally oversimplified cost-effectiveness model comparing the three arms of GOG218 study, Cohn et al. demonstrated that the addition of bevacizumab to the adjuvant management of patients with advanced ovarian cancer is not cost effective and treatment with maintenance bevacizumab, while improving PFS, is associated with both direct and indirect costs [125]. Thus, the optimal duration of maintenance treatment with bevacizumab will also have to be evaluated, and pharmacoeconomic considerations will have to be addressed [9].

### 7.4. Other Challenges

Despite the advances, some other critical challenges in both clinical-pathologic and preclinical investigations are still ahead [11]. Lack of predictive markers and accurate predictors of therapeutic efficacy seems to be a major challenge of anti-VEGF therapy in ovarian cancer [9, 89, 126]. To date, there are no predictive biomarkers for response to bevacizumab which means that there is no preselection of patients who might benefit from therapy [89]. IL-8 and VEGF polymorphisms have been suggested as potential markers of clinical outcome after bevacizumab-based chemotherapy in refractory ovarian cancer [126]. Retrospective studies will be performed on blood and tumor to study VEGF levels and other angiogenic markers in blood and tumor to try and identify which patients might benefit from bevacizumab [89]. Clinically validated biomarkers by restricting the VEGF-targeted therapy to the selected patients will also reduce the rate of relevant complications, in particular gastrointestinal perforations. Since large placebo-controlled phase III trials are still missing, well-designed trials in which potentially important clinical effects of anti-VEGF agents are not ignored are highly required [9]. These clinical benefits may manifest as prolongation of survival, delay in progression of disease, reduction of tumor burden, alleviation of symptoms associated with the disease, and minimization of toxicities associated with the treatment of the disease [127]. Also, it has been learned that the mere presence of a particular target does not guarantee the therapeutic benefit of the relevant targeted therapy, and, due to the multiplicity and redundancy of the pathways, it is unlikely that inhibition of a single cascade will be highly effective [33, 128]. A better understanding of the relevant signaling pathways, targeting horizontal and vertical pathways, and unveiling the underlying mechanisms of resistance and complications are among the goals of the future studies [11, 98].

## 8. Conclusion

VEGF is one of the most potent effectors of physiologic and pathologic angiogenesis. The pathophysiology of ovarian cancer is extremely angiogenesis-dependent [9]. Highly expressed in ovarian cancer, VEGF represents an attractive therapeutic target and VEGF inhibitors promise to be of significant value in the treatment of ovarian cancer. Preclinical and clinical studies further support the utility of these approaches. The most promising and widely explored bevacizumab has been used in many clinical studies either as single agent or in combination with chemotherapy in women with resistant or recurrent ovarian cancer. However, lack of accurate predictors of therapeutic efficacy, primary or secondary resistance to the treatment, complications and side effects of the therapy, likely divergent effects of anti-VEGF therapy on primary tumor growth and metastasis, and pharmacoeconomic concerns are the major struggles in the clinical use of VEGF inhibitors in malignancies. Some of the goals to be targeted in future studies include improvement in clinical trial design so that potentially important clinical effects of these agents are not ignored, a more detailed comprehension of VEGF inhibition pathways and discernment of optimal combination therapy. Further investigations are warranted to identify predictive biomarkers required for “individualization” of VEGF-targeted therapy, and to precisely clarify the mechanisms underlying the complications of the treatment.

## Figures and Tables

**Table 1:** Anti-VEGF agents in clinical development for ovarian cancer.

Mechanism(s) of action	Drug	Molecular target(s)
VEGF ligand binders	Bevacizumab	VEGF A (all isoforms)
	Aflibercept (VEGF trap)	VEGF A and B, PlGF

VEGF receptor tyrosine kinase inhibitors	Ramucirumab	VEGFR2
	Cediranib	VEGFR1-3, c-Kit, PDGFR-*β*
	Semaxanib	VEGFR2

Multiple-receptor tyrosine kinase inhibitors	Sunitinib	VEGFR1-3, Flt-3, PDGFR-*α*, PDGFR-*β*, c-Kit, CSF-1R, RET
	Sorafenib	VEGFR1-3, PDGFR-*β*, Flt-3, c-Kit, Raf-1
	Vatalanib	VEGFR1-3, PDGFR*β*, c-Kit, c-Fms
	Intedanib (BIBF1120)	VEGFR1-3, PDGFR-*α*, PDGFR-*β*, FGFR1-3
	Pazopanib	VEGFR1-2, PDGFR-*β*, c-Kit
	Motesanib	VEGFR1-3, PDGFR, c-Kit
	Vandetanib	VEGFR2-3, EGFR
	AEE788	VEGFR, EGFR

VEGF: vascular endothelial growth factor, PlGF: placenta growth factor, VEGFR: vascular endothelial growth factor receptor, PDGFR: platelet-derived growth factor receptor, CSF-1R: colony stimulating factor 1 receptor, EGFR: epidermal growth factor receptor.

**Table 2:** Clinical trials of single-agent therapy with bevacizumab in ovarian cancer.

Trial number	Phase	Stage of the disease	Number of patients	CR	PR	MPFS (m)	MOS (m)
Completed							
NCT00022659 (GOG170D) [37]	II	Persistent or recurrent EOC or PPC	62	3%	18%	4.7	17
NCT00097019 (AVF2949g) [38]	II	Recurrent EOC or PSC	44	0	15.9%	4.4	10.7
Ongoing							
NCT00866723(08-323)*	II	Relapse after bevacizumab maintenance therapy	32	N/A	N/A	N/A	N/A

EOC: epithelial ovarian cancer, PPC: primary peritoneal cancer, PSC: peritoneal serous carcinoma, CR: complete response, PR: partial response, MPFS: median progression-free survival, MOS: median overall survival, m: months, N/A: nonaccessible.

*Accessed from http://www.clinicaltrials.gov/ on April 18, 2011.

**Table 3:** Completed clinical trials of bevacizumab combined with chemotherapy in ovarian cancer.

Trial number	Phase	Chemotherapy	Stage of the disease	Number of patients	Outcomes
NCT00127920 (AV53206s) [39]	II	Carboplatin + paclitaxel	Newly diagnosed stage III/IV	20	CR: 30% PR: 50%
NCT00072566 (NCI-5789) [40]	II	Metronomic cyclophosphamide	Platinum-sensitive recurrent	70	CR: 0 PR: 24% MPFS: 7.2 (m) MOS: 16.9 (m)
NCT00129727 (OVCA) [41]	II	Carboplatin + paclitaxel + bevacizumab + maintenance bevacizumab	Newly diagnosed stage ≥ IC	62	CT: CR: 56% PR: 22% Ca-125: CR: 89% PR: 7%
NCT00343044 (3040200, AVF3648s)*	II	Topotecan	Platinum-resistant recurrent EOC, PPC, FTC	N/A	N/A

CR: complete response, PR: partial response, MPFS: median progression-free survival, m: months, MOS: median overall survival (months), EOC: epithelial ovarian cancer, PPC: primary peritoneal cancer, FTC: fallopian tube cancer.

*Accessed from http://www.clinicaltrials.gov/ on April 18, 2011.

**Table 4:** Ongoing clinical trials of bevacizumab combined with chemotherapy in ovarian cancer.

Trial number	Phase	Chemotherapy	Stage of the disease	Number of patients (estimated)
NCT00127920 (AV53206s)	II	Carboplatin + paclitaxel + bevacizumab	Newly diagnosed stage III/IV	20
NCT00296816 (TEACO)	II	Oxaliplatin + docetaxel + bevacizumab	Newly diagnosed stage IB-IV	145
NCT00511992 (AVF3953)	II	Paclitaxel + cisplatin + bevacizumab followed by bevacizumab	Newly diagnosed stage II-III	20
NCT00588237 (06-064)	II	Paclitaxel + cisplatin + bevacizumab	Initial treatment of optimal stage II or III (adjuvant)	42
NCT00267696 (2005CO073)	II	Gemcitabine + carboplatin + bevacizumab	Platinum-sensitive recurrent	45
NCT00698451 (CR015094)	II	Carboplatin + liposomal doxorubicin + bevacizumab	Platinum-sensitive recurrent	54
NCT00418093 (04-356)	II	Oxaliplatin + gemcitabine + bevacizumab	Platinum-sensitive recurrent	40
NCT00868192 (08-0508)	II	Pemetrexed + bevacizumab	Recurrent having failed platinum- and taxane-based regimens	25
NCT00504257 (MCC-14920, MCC-105366c)	II	Docetaxel + bevacizumab	Platinum-resistant recurrent	44
NCT00744718 (2008-000878-20, S-20080033)	II	Carboplatin + bevacizumab	Platinum-resistant recurrent	30
NCT00846612 (06-948, AVF3910s)	II	Liposomal doxorubicin + bevacizumab	Platinum-resistant recurrent	48
NCT00856180 (08-148)	II	Cyclophosphamide + bevacizumab	Platinum-resistant recurrent	20
NCT00407563 (ALSSOPR0501)	II	Abraxane (protein-bound paclitaxel ) + bevacizumab	Platinum-resistant recurrent	48
NCT00937560 (MO22225)	II	Carboplatin + paclitaxel + bevacizumab	Previously untreated, but initial surgery	188
NCT00583622 (2007-0368)	II	Gemcitabine + docetaxel + melphalan + carboplatin + bevacizumab	Second or later complete remission, or untreated or refractory relapse to platinum treatment or lack of response to salvage treatment	40
NCT00483782 (ICON7)	III	Carboplatin + paclitaxel ± bevacizumab	Newly diagnosed	1520
NCT00262847 (GOG218)	III	Carboplatin + paclitaxel versus carboplatin + paclitaxel + bevacizumab ± maintenance bevacizumab	Newly diagnosed, previously untreated stage III or IV	2000
NCT00565851 (GOG213)	III	Carboplatin + paclitaxel ± bevacizumab followed by bevacizumab and secondary cytoreduction surgery	Platinum-sensitive recurrent	660
NCT00434642 (OCEANS, AVF4095g)	III	Carboplatin + gemcitabine ± bevacizumab	Platinum-sensitive recurrent	487
NCT00652119 (2007-0223)	N/A	Carboplatin + paclitaxel + bevacizumab	Newly diagnosed stage III/IV	46

*Accessed from http://www.clinicaltrials.gov/ on April 18, 2011.

**Table 5:** Clinical trials for VEGF trap in ovarian cancer.

Trial	Phase	Stage of the disease	Results
*Single agent*			
NCT00327171 (ARD6122, AVE0005)—Completed	II	Platinum-resistant and topotecan and/or liposomal doxorubicin-resistant advanced ovarian cancer	Preliminary results from 162 randomized patients showed a partial response of 11%. [53]
NCT00396591 (ARD6772)—Completed	II	Platinum-resistant and topotecan and/or liposomal doxorubicin-resistant advanced ovarian cancer with recurrent symptomatic malignant ascites	First results demonstrated the efficacy of two weekly IV aflibercept in prolonging the time to repeat paracentesis in eight out of ten evaluable patients. [54]
NCT00327444 (EFC6125)—Completed	II/III	Platinum-resistant and topotecan and/or liposomal doxorubicin-resistant advanced ovarian cancer with recurrent symptomatic malignant ascites	Results are awaited

*Combination with chemotherapy *(docetaxel)			
NCT00436501 (MDA-2006-0329)—ongoing	I/II	Recurrent or persistent epithelial ovarian, primary peritoneal, or fallopian tube cancer	Ongoing

IV: Intravenous, VEGF: Vascular endothelial growth factor.

*Accessed from http://www.clinicaltrials.gov/ on April 18, 2011.
